# Posttraumatic Growth, Centrality of Event, Trauma Symptoms and Resilience: Profiles of Women Survivors of Intimate Partner Violence

**DOI:** 10.1177/08862605211050110

**Published:** 2021-10-17

**Authors:** Aistė Bakaitytė, Goda Kaniušonytė, Rita Žukauskienė

**Affiliations:** 1 Institute of Psychology, 87247 Mykolas Romeris University, Vilnius, Lithuania

**Keywords:** intimate partner violence, posttraumatic growth, centrality of event, trauma symptoms, resilience, mental health and violence

## Abstract

The current study used a person-oriented approach to investigate (a) potential distinctive groups of women survivors of IPV based on their posttraumatic growth (PTG), centrality of event, resilience, and posttraumatic stress symptoms (PTSS) patterns, and (b) examine the role of sociodemographic (age, education, work status) and violence related (physical and emotional violence, time since last violence episode, psychological help) factors in distinguishing these groups. The study sample consisted of 421 women survivors of IPV, and latent profile analysis revealed four profiles: “negative impact” (11% of the sample), “positive growth” (46%), “low impact” (18%), and “distressed growth” (25%). Women age, education, received psychological help, frequency of physical and emotional violence, and time since last violence incident significantly distinguished some of the indicated profiles from each other. Findings of this study contribute to the existing literature by identifying different responses to IPV and investigating some of the theoretical assumptions that had not been comprehensively analyzed in the IPV literature. Limitations of the study and implications for future research are discussed.

## Introduction

Intimate partner violence (IPV) is conceptualized as physical, sexual, economic, and/or psychological harm caused by a current or former intimate partner (World Health Organization [WHO], 2017). Findings from the analysis of the 141 studies on intimate partner violence show that globally, in 2010, 30% of women aged 15 and over have experienced physical and/or sexual violence during their lifetime (Devries et al., 2013; WHO, 2017). However, in Lithuania, IPV prevalence rates are even higher, as a national representative survey (*N* =1173) revealed that more than a half of the surveyed women (51.2%) had suffered from some type of IPV at least once in their lifetime (Žukauskienė et al., 2021).

IPV is a challenging and traumatizing experience, affecting psychological well-being and the victim’s overall functioning. It is well documented that violence disrupts victims’ social and daily functioning, leading to mental health problems, however, an increasing number of studies have demonstrated that some survivors of IPV are also experiencing posttraumatic growth (PTG; Cobb et al., 2006; Ulloa et al., 2015; Valdez & Lilly, 2015). Factors related to PTG are conceptualized in Tedeschi and Calhoun’s (1996) model and are studied in the context of various traumatic experiences. However, IPV has specific dynamics and differs from other traumatic experiences: IPV may occur over an extended period, and an imbalance of power in their relationships make women, for the most part, dependent on their violent partners (Ulloa et al., 2015). Therefore, it is important to study PTG in samples of IPV survivors to determine whether PTG occurs in the same or a similar way as in other traumatic experiences. The current study used a person-oriented approach (a) to investigate potential distinctive groups of women survivors of IPV based on their PTG, the centrality of event, resilience, and posttraumatic stress symptom (PTSS) patterns, and (b) to examine the role of sociodemographic and violence-related factors in distinguishing these groups.

### The Process of Posttraumatic Growth

Posttraumatic growth (PTG) is defined as positive psychological changes in the aftermath of traumatic experiences, and these changes can be experienced in three broad categories: relationships with others, philosophy of life, and view of the self (Tedeschi & Calhoun, 1996). PTG can be understood as a process and an outcome. To explain PTG as a process, the authors used an earthquake analogy (Tedeschi & Calhoun, 2004), where traumatic events, like an earthquake, can shatter or destroy person’s schematic structures which provided the basis for their world view, decision-making, and meaning before trauma. When these schematic structures are shattered or destroyed, a person experiences great distress but through cognitive processing and restructuring, schematic structures can be rebuilt based on what was destroyed and what is left, similar to the manner in which houses, and cities are rebuilt after an earthquake (Tedeschi & Calhoun, 2004). This analogy is a good one because it shows that the PTG process is not only positive, it requires suffering and struggle through traumatic experience until some positive changes can be reached.

Tedeschi and Calhoun introduce PTG as a process with a theoretical model of PTG, which is constantly revised based on new research in the field, with the most recent version of the PTG model presented in 2018 (Tedeschi et al., 2018). The process of PTG first goes through the centrality of event, which represents how central to a person’s identity the traumatic experience is. If the traumatic event is perceived as central, the PTG process takes place and cognitive processing leading to PTG may begin. Conversely, if the traumatic experience can be integrated into a person’s identity and core beliefs are not challenged, emotional distress is experienced in a way that produces resilience without great personal change or growth (Tedeschi et al., 2018). It is important to note, that perceived positive changes do not necessarily eliminate emotional distress which is often experienced by posttraumatic stress symptoms (PTSS). This means that even when positive changes are experienced, negative emotional consequences (such as PTSS) can also be present (Bensimon, 2012; Triplett et al., 2012). Therefore, the model of PTG indicates that traumatic experiences can be differently perceived by various people, which in turn, can have different outcomes for them.

Centrality of event refers to the extent in which the event is a turning point in person’s life story, and in general, is significant to a person’s identity, leading to either the validation or reconsideration of current beliefs, values, and world view (Berntsen & Rubin, 2006). Evidence in the literature suggests that centrality of event can be a “double-edged sword,” leading to both negative (e.g. PTSD, depression) and positive (e.g. growth) outcomes (Boals & Schuettler, 2011). As noted earlier, if the event is perceived as significant to person’s life story and challenges or disrupts core beliefs, it provides the potential for the PTG process to occur. People experience traumatic events differently, and the same event can have different meanings for different people (Boals et al., 2010). Considering the theoretical model of PTG, centrality of event may be one of the main things distinguishing different people and their perception of potentially traumatic events, that in turn can have different outcomes. Therefore, we decided to include centrality of event in our study.

Early research on resilience started with a focus on children growing up in adverse circumstances, but in the last two decades there has been an emergence of resilience research in adults (Infurna & Jayawickreme, 2019). Even though resilience has been studied for a few decades now, there is still no universally defined concept for it. Some researchers define resilience as the absence of negative consequences of traumatic events (e.g., depression, PTSD), others see it as a personality characteristic (rather stable) or as a process (Mukherjee & Kumar, 2017). Although, some authors consider resilience and PTG to be the same constructs (Sattler et al., 2014), Tedeschi et al. (2018) distinguish these two concepts indicating that resilience in general represents the ability to “bounce back” to pre-trauma levels of functioning while PTG goes beyond this level of functioning resulting from struggle with difficult circumstances caused by trauma. In the context of PTG theoretical model, resilience plays dual role, and that can explain mixed findings in the literature where some researchers find negative relation between PTG and resilience (Levine et al., 2009), and others find a positive relation (Bensimon, 2012; Oginska-Bulik, 2015). On one hand, if a person is highly resilient before the traumatic event, it is likely that they will not engage in the after-trauma cognitive processing that is needed for the PTG process (Westphal & Bonanno, 2007). This means that highly resilient people may not be that prone to experience PTG because of their ability to “bounce back” quickly after difficult circumstances without challenging their core beliefs. On the other hand, if a person struggles and goes through cognitive processing of their experience, resilience can be enhanced after experiencing PTG as a result of newly gained strengths and notions of surviving (Tedeschi & Blevins, 2017). Considering these mixed findings and the plausible theoretical explanations, there is a reason to suspect that different groups of women will respond differently to intimate partner violence as a consequence of their resilience level.

### Sociodemographic and Violence-Related Factors Associated with Posttraumatic Growth

A limited number of studies investigate the relation between sociodemographic factors and posttraumatic growth (PTG) in women survivors of intimate partner violence (IPV). In those limited studies, older age is often related to higher levels of PTG (Grace et al., 2015; Grubaugh & Resick, 2007). Considering education, studies reveal mixed findings. In some studies, PTG is positively related to education level (Grace et al., 2015; Wang et al., 2014), and in others, including sample of IPV survivors, this relation is negative (Grubaugh & Resick, 2007; Koutrouli et al., 2012; Žukauskienė et al., 2019). Although we could not find any study of IPV survivors that examined relation between work status and PTG, studies in other samples find positive associations, where employed people tend to experience higher levels of PTG (Bellizzi & Blank, 2006; Xu & Wu, 2014). Considering the scarcity of research on sociodemographic factors and PTG in IPV survivors, and their importance in other samples, we decided to include women’s age, education, and work status as predictors in our analysis hoping that these factors could help us better understand distinguished profiles.

There are a few important violence-related factors that may be related to PTG. Tedeschi et al. (2018) suggest that PTG requires some time to occur. Studies with women survivors of IPV confirmed that more time that has elapsed after the last violent experience is related to greater PTG (Bakaitytė at al., 2022; Doane, 2011). Furthermore, studies have shown that more frequent violence is associated with greater PTG (Cobb et al., 2006; Doane, 2011; Žukauskienė et al., 2019), but most studies investigating IPV focus on physical or combined indicators of violence (including all types of violence together). However, Hill et al. (2009) argue that psychological violence may be more detrimental to women’s mental health than physical violence. This suggests that different types of violence can also be differently related to IPV outcomes, such as PTG, and it may be beneficial to examine physical and psychological violence separately. For this reason, we decided to separate frequency of physical and psychological violence and included them in our analysis as predictors of distinguished profiles.

Another important factor associated with PTG is help received after IPV experience. The theoretical model of PTG posits growth as a consequence of cognitive processing that leads to rebuilt schematic structures shattered by traumatic events (Tedeschi et al., 2018). Therefore, professional psychological help may be crucial for successful cognitive processing leading to PTG (Hassija & Turchik, 2016). Keeping this in mind, we think that psychological help may be a factor that helps us better understand analyzed profiles, and for this reason we included it as a predictor in our analysis.

### Current Study

The majority of research of posttraumatic growth (PTG) to date has been variable-centered, examining relations within exposure to intimate partner violence (IPV), PTG and its’ factors presented in theoretical model (Bakaitytė, et al., 2022; Boals, et al., 2010). The heterogeneity within women survivors of IPV with regards to different combinations of variables related to PTG into clearly distinctive patterns has not been sufficiently addressed. This may partially be attributed to the lack of sufficiently large samples and/or associated with a pre-dominant variable-focused rather than person-oriented approach (Bogat et al., 2005; von Eye & Bergman, 2003). The theoretical model of PTG (Tedeschi et al., 2018) suggests that there may be different responses to traumatic events, such as IPV. Therefore, in the present study, we aim to (a) explore potential distinctive groups of women survivors of IPV based on their posttraumatic growth, event centrality, resilience, and PTSS patterns, and (b) examine the role of sociodemographic (age, education, work status) and violence-related (frequency of physical and emotional violence, time since last violent event, psychological help) factors in distinguishing these groups. In doing so, we are using propositions from person-oriented research that indicate that distinct subgroups existing in a sample with substantively meaningful subgroup characteristics (Bogat et al., 2005; von Eye & Bergman, 2003).

## Method

### Participants and Procedures

Data sample for this study was combined from two samples. In the first sample, 221 women from different regions of Lithuania who sought help from women’s shelters, social support centers, and counseling psychologists were asked to participate in this study. Questionnaires were administered both on paper and online. In the second sample, multistage stratified quota sampling was used. Data collection was completed by 37 interviewers (only women), who collected data from different regions of Lithuania by going to the homes of potential study participants using the snowball method or information from local social workers. Questions about intimate partner violence (IPV) were administered first to identify IPV survivors. If respondent indicated at least one physical or sexual violence incident, or at least three psychological or economic violence incidents from current or former intimate partner, she was considered an IPV survivor and other questionnaires were presented. Considering that psychological and economic violence are more nuanced and some of the items of these subscales of violence may also have reflected one-time conflicts in relationships (e.g. “Ignored, did not speak, did not answer questions,” “Demanded to tell me how and where I spend my money”), we have introduced stricter inclusion criteria for frequency of these types of violence. Overall, 200 women with the history of IPV participated in this study (second sample). Questionnaires were administered on paper. All participants were asked if they felt safe to fill in the questionnaires at home. In both samples, the questionnaires were identical and were administered in the same order. Data in both samples was gathered under the study on identity and posttraumatic growth (PTG) in female survivors of intimate partner violence (INTEGRO). This study has been approved by the Mykolas Romeris University, Institute of Psychology.

The total sample consisted of 421 women. The mean age of the participants was 41.70 (SD = 11.96). Less than a half (40.6%) of the women were currently living with a partner, 32.1% were single, 19.7% had a partner but were not living together, 6.4% were involved in episodic relationships, and 1.2% declined to report their relationship status. More demographic variables are presented in Table 1. The IPV-related sample characteristics are presented in Table 2.

**Table 1.** Sample characteristics.

Characteristics	*n* (%)
Age	
17–24	21 (5.0)
25–34	102 (24.2)
35–44	146 (34.7)
45–54	81 (19.2)
55+	70 (16.6)
No response	1 (0.2)
Education	
Primary (up to Grade 4)	6 (1.4)
Lower secondary (up to Grade 10)	60 (14.3)
Secondary (up to Grade 12)	123 (29.2)
Higher education (Junior College)	74 (17.6)
Higher education (College)	64 (15.2)
Higher education (University)	94 (22.3)
Work status	
Employed	279 (66.3)
Studying	13 (3.1)
Unemployed/not studying	115 (27.3)
No response	14 (3.3)
Place or residence	
City (>50.000 residents)	139 (33)
Town (2.000–50.000 residents)	188 (44.6)
Village (<2.000 residents)	92 (21.9)
No response	2 (0.5)

**Table 2.** IPV-related characteristics.

	*n* (%)
Forms of IPV in the sample	
Psychological violence	398 (94.5)
Economical violence	315 (74.8)
Physical violence	343 (81.5)
Sexual violence	245 (58.2)
Relationship status with the perpetrator	
Living with the perpetrator	141 (33.5)
Currently in divorce process	79 (18.8)
No longer in a relationship with perpetrator	192 (45.6)
No response	9 (2.1)
Time since last violence incident	
Less than a week	24 (5.7)
More than a week	27 (6.4)
More than a month	58 (13.8)
More than a half year	50 (11.9)
More than a year	39 (9.3)
More than 2 years	68 (16.2)
More than 5 years	66 (15.7)
More than 10 years	44 (10.5)
More than 20 years	33 (7.8)
No response	12 (2.9)
Received psychological help	
Yes	164 (39)
No	241 (57.2)
No response	16 (3.8)

### Measures

Posttraumatic growth (PTG) was measured with the Short Form of Posttraumatic Growth Inventory (PTGI-SF; Cann et al., 2010; Tedeschi & Calhoun, 1996) which consists of 10 items. Participants rated the items (e.g. “I changed my priorities about what is important in life”) on a 6-point Likert-type scale ranging from 0 (*I did not experience this change*) to 5 (*I experienced this change to a very great degree*). The Cronbach’s alpha of the scale was .95. A confirmatory factor analysis (CFA) indicated an acceptable structural validity of the scale, *χ*^2^ (45) 2015.53, Tucker-Lewis index (TLI) = .96, comparative fit index (CFI) = .97, root mean square error of approximation (RMSEA) = .07. Here and later, the model fit was evaluated following the recommendations provided by Little (2013): TLI/CFI values higher than .90 indicated an acceptable fit and values higher than .95 indicated a very good fit; RMSEA values below .08 indicated acceptable fit and values below .05 represent a good fit. Factor scores of the scale were used for the latent profile analysis.

Centrality of events was measured with the Centrality of Events Scale (CES; Berntsen & Rubin, 2006) which consists of seven items. Items (e.g. “This event was a turning point in my life”) were rated on a 5-point Likert-type scale ranging from 1 (*Totally disagree*) to 5 (*Totally agree*). Cronbach’s alpha of the scale was .89. Results of CFA indicated a very good structural validity of the scale, *χ*^2^ (21) 924.331, TLI = .98, CFI = .99, RMSEA = .05. Factor scores of the scale were used for the final analysis.

Resilience was measured with the 14-item Resilience Scale (Wagnild and Young, 1993). Participants rated items (e.g. “I usually manage one way or another”) on a 7-point Likert-type scale ranging from 1 (*Strongly disagree*) to 7 (*Strongly agree*). Cronbach’s alpha of the scale was .93. CFA indicated an acceptable structural validity of the scale, *χ*^2^ (91) 2034.77, TLI = .93, CFI = .94, RMSEA = .06. Factor scores of the scale were used for final analysis.

Posttraumatic stress symptoms were measured with Impact of Event Scale-Revised (IES-R) (Weiss & Marmar, 1996) which consists of 22 items. Participants rated items (e.g. “Any reminder brought back feelings about it”) on a 5-point Likert-type scale ranging from 0 (*Not at all*) to 4 (*Extremely*). Cronbach’s alpha of the total scale was .96. CFA indicated an acceptable structural validity of the scale, *χ*^2^ (231) 5431.99, TLI = .91, CFI = .92, RMSEA = .07. Factor scores of the total scale were used for final analysis.

Frequency of different forms of intimate partner violence (IPV) were assessed with a 21-item checklist, developed by the authors of this manuscript. Development of the checklist was based on the Composite Abuse Scale (Ford-Gilboe et al., 2016) and the Scale of Economic Abuse (Adams et al., 2008). The checklist measures frequency of four types of violence: *psychological* (8 items, e.g. “Insulted, humiliated (e.g. told you that you are not good enough, ugly, stupid and etc.”), *economic* (5 items, e.g., “Took money from you purse or bank account without your permission”), *physical* (5 items, e.g., “Beat you by hand or fist”), and *sexual* (3 items, e.g., “Physically forced you to have sexual intercourse when you did not want to”). Participants indicated each behavior on an 8-point Likert-type scale ranging from 0 (*Never happened to me*) to 7 (*Happens to me every day*). CFA indicated an acceptable structural validity, *χ*^2^ (177) 483.19, TLI = .90, CFI = .91, RMSEA = .06. For this study, the psychological and economic violence and physical and sexual violence subscales were combined to indicate *emotional* and *physical violence*. The Cronbach’s alpha coefficients for emotional and physical violence were .90 and .86, respectively. The mean scores of the subscales were used for analysis.

Single items measured additional variables of age, place of residence, education, work status, received psychological help and time after last violence incident. For the multinomial logistic regression, the dummy variables for work status and the time since the last violence incident were created. For work status, we created two dichotomized variables: Employed variable (0—unemployed/not studying and studying; 1—employed) and unemployed/not studying (0—employed and studying; 1—unemployed/not studying). The time since last violence incident was dichotomized as follows: 0—violence experienced less than 2 years ago and 1—violence experienced more than 2 years ago.

### Data Analysis

In the current study, we aimed to explore potential distinctive groups of women survivors of intimate partner violence (IPV) based on their posttraumatic growth (PTG), centrality of event, posttraumatic stress symptoms, and resilience patterns, and to examine the role of demographic and violence-related factors in predicting these groups. All Latent Profile Analyses (LPA) were conducted using Mplus 8.4 (Muthén & Muthén, 1998) with full information maximum likelihood estimation. To identify the best LPA solution, a series of LPA models, starting with one profile, were conducted, and evaluated. To decide on the number of profiles, we followed recommendations for Latent Class Analysis (LCA; Nylund et al., 2007). We used several criteria: Akaike Information Criterion (AIC) which should be lower than solution with k-1 profiles; a statistically significant *p*-value of the Lo, Mendell, and Rubin (LMR) test, which compares models and indicates when additional profiles are not improving fit of the model; high entropy values (0.80) indicate that each profile group is unique (Nylund et al., 2007). Additionally, we examined substantive meaningfulness of the latent profiles.

To examine group differences in the resulting profiles, we used three-step approach (Asparouhov & Muthén, 2014). In a first step, only latent profile indicator variables were used to estimate latent profile model. In the second step, the most likely profile variable was created based on latent profile distribution obtained in the first step. Finally, the most likely profile was regressed on predictor variables by performing multinomial logistic regression. After estimating the best profile solution, for the subsequent steps we used automatic R3STEP procedure available in Mplus 8.4.

To determine whether the data were missing at random, we conducted a normed χ^2^ (χ^2^/df ratio) test. There is agreement that a value less than 2.0 indicates that data were missing at random, and that maximum likelihood techniques were appropriate for use (Bollen, 1989). The normed χ^2^ value was 1.20. Using full information maximum likelihood (FIML, Full Information Maximum Likelihood available in Mplus), analyses were conducted using all available data from the total sample (*N* = 421). Whereas, R3STEP procedure in Mplus utilizes listwise deletion if the participant has missing data in the covariates, some participants (*n* = 69) were excluded from covariate analysis.

## Results

### Preliminary Analysis

Bivariate correlations, means, and standard deviations of the study variables are reported in Table 3. As can be seen, posttraumatic growth (PTG) is positively related to centrality of event and resilience, and posttraumatic stress symptoms (PTSS) are negatively associated with resilience and positively with centrality of event. PTG is unrelated to PTSS and resilience is unrelated to centrality of event.

**Table 3.** Correlations among study variables and descriptive statistics.

	1	2	3	4
1. Posttraumatic growth	—			
2. Resilience	.38**	—		
3. Centrality of event	.37**	.09	—	
4. Posttraumatic stress symptoms	.03	−.25**	.39**	—
M	2.96	5.02	3.19	1.19
SD	1.35	0.87	1.02	0.88

**p < .001.

### Profiles of Posttraumatic Growth, Resilience, and Centrality of Event

**Figure 1.**
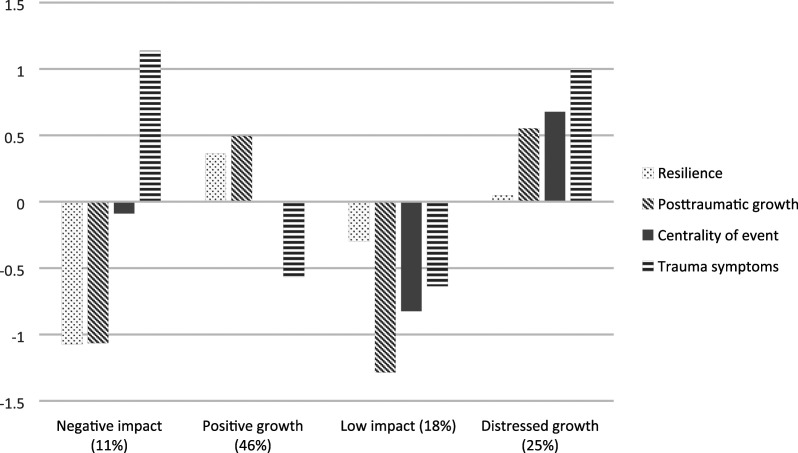
Latent profiles based on factor means of study variables.

To explore potential distinctive groups of women survivors of intimate partner violence (IPV), Latent Profile Analysis (LPA) was conducted. Table 4 presents the goodness-of-fit information for LPA models with one–five groups. The 4-profile model fitted the data best; although the 5-profile model had lower AIC values, LMR results supported the 4-profile model, which also had a slightly better Entropy value than the 5-profile solution. Mean factor scores of resilience, posttraumatic growth (PTG), centrality of event, and posttraumatic stress symptoms (PTSS) in each profile are presented in Figure 1. The first profile (*n* = 45; 11%) is characterized by low levels of resilience and PTG, medium levels of centrality of event, and high levels of PTSS, and was named as *negative impact* profile. The second, *positive growth*, profile (*n* = 194; 46%) is distinguished by higher than average levels of resilience and PTG, medium levels of centrality of event, and low levels of PTSS. The third, *low impact*, profile (*n* = 76; 18%) represents low levels of all profile indicators. And finally, the fourth, *distressed growth*, profile (*n* = 106; 25%) is characterized by medium levels of resilience and high levels of PTG, centrality of event and PTSS.

**Table 4.** Model fit statistics for latent profile analysis.

Classes	Log likelihood	AIC	Entropy	LMR *p*-value	Smallest class *n* (%)
1	−2284.789	4585.578	—		
2	−2194.436	4414.873	.81	.000	122 (39)
3	−2170.118	4376.236	.73	.588	71 (17)
**4**	**−2114.114**	**4274.227**	**.81**	**.010**	**45 (11)**
5	−2096.986	4248.772	.80	.335	25 (6)

*Note.* AIC, Akaike Information Criterion, LMR, Lo, Mendell, and Rubin test.

### Socio-Economical and Violence-Related Predictors of Latent Profiles

To examine the role of demographic and violence-related factors in predicting latent profiles, multinomial logistic regression was conducted with the *distressed growth* group as a reference group, and the results are presented in Table 5. Results indicated that, as age increases, the odds of being in the *low impact* profile versus the *distressed growth* profile decreases. This means that the *distressed growth* profile consists of more older women than the *low impact* profile. For education, women with higher education tended to belong to the *distressed growth* profile more than to the *negative impact* profile. Work/studying statuses were not related to either of the profiles.

**Table 5.** Covariate analysis results for the four-profile model.

Variable	Negative impact (*n* = 45)	Positive growth (*n* = 194)	Low impact (*n* = 76)
Age	−0.040	−0.032	−0.061*
Education	−0.471*	−0.280	−0.059
Psychological help	−2.242*	−0.777	−1.666*
Employed	−2.673	−1.263	−3.312
Unemployed/not studying	−2.134	−1.405	−1.760
Physical violence	−0.431	−0.255	−0.974*
Emotional violence	−0.171	−0.202	−0.839*
Last violence incident more than 2 years ago	−1.182*	1.090*	−1.143*

*Note. Distressed growth* (*n* = 106) profile served as the reference group. *N* = 352.

*p < .05.

For women who received psychological help, the odds of being in the *negative impact* and *low impact* profiles versus the *distressed growth* profile decreases, indicating that the *distressed growth* profile consists of more women who received psychological help than the *low impact* and *negative impact* profiles. Physical and emotional violence was experienced by women in the *distressed growth* profile more frequently than in the *low impact* profile. Women who experienced their last violence incident more than 2 years ago were more likely to belong to the *distressed growth* profile compared to the *negative impact* and *low impact* profiles. The *positive growth* profile consisted of more women who experienced their last violence incident more than 2 years ago compared to the *distressed growth* profile.

## Discussion

The purpose of the current study was (a) to explore potential distinctive groups of women survivors of IPV based on their posttraumatic growth (PTG), centrality of event, resilience, and posttraumatic stress symptoms (PTSS) patterns, and (b) to examine the role of sociodemographic (age, education, work status) and violence-related (physical and emotional violence, time since last violence incident, psychological help) factors in predicting these groups. By examining relationships at the person level rather than the variable level, person-oriented approach (Bergman & Magnusson, 1997) enabled us to distinguish common patterns of characteristics that apply to one subgroup and distinguish it from another subgroup. Overall, our analysis revealed four groups of women differing by PTG, centrality of event, resilience, and PTSS patterns. Also, some of the identified profiles were distinguished by sociodemographic and violence-related factors.

### Profiles of Women Survivors of Intimate Partner Violence

In addressing our first research question, we found four different profiles of women survivors of intimate partner violence (IPV) based on their posttraumatic growth (PTG), centrality of event, resilience, and posttraumatic stress symptoms (PTSS) patterns.

The largest group of women (46%) were those that displayed above-average levels of PTG and resilience, medium levels of centrality of event, and lower than average levels of PTSS. In a light of the theoretical model of PTG (Tedeschi et al., 2018), this group of women display growth patterns as they perceive their IPV experience as central (although these levels are average), and their resilience and PTG levels are high. For this reason, we named this group the *positive growth* group, where *positive* represents their low levels of PTSS. The second largest group of women (25%) was characterized by above-average levels of PTG, centrality of event and PTSS, and average levels of resilience. This group of women also displayed growth patterns but with high levels of PTSS, so we named it the *distressed growth* group. A third group of women (18%) display low levels of all indicators, meaning that these women did not perceived their IPV experience as central to their identity, they did not experienced PTG, their resilience levels were below average, and they did not experience PTSS. In general, this group represents women that did not appear to be affected by their IPV experience with regards to the included measures, so we named it the *low impact* group. Finally, the smallest group of women (11%) was distinguished by low levels of resilience and PTG, average levels of centrality of event, and high levels of PTSS. These findings indicate that similar to the *positive growth* group these women perceive their IPV experience as central to their life stories, however, their recovery process is not similar to those in the *positive growth* group, as they express PTSS without positive changes or resilient response. For this reason, we named this group the *negative impact* group.

In a way, our results are consistent with Tillery et al. (2016) study where similar three profiles, based on PTSS and PTG patterns, were found in youth with cancer. Their results revealed a group with high growth and low PTSS, a group with low PTG and low PTSS, and a group with high PTSS and low PTG, which in part could support our *positive growth*, *low impact*, and *negative impact* groups. Considering this, it can be assumed that our and Tillery et al. (2016) study captures some common aspects of responses to traumatic experiences in different groups of people.

### Sociodemographic and Violence-Related Predictors

To address our second research question, we included sociodemographic (age, education, and work status) and violence-related (time since last violence incident, frequency of violence, and psychological help) predictors of distinguished profiles in our analysis. We will describe each of the profiles comparing them with the *distressed growth* profile because it served as a reference group in the analysis. This group was chosen as reference group because it best fit theoretical model where posttraumatic growth (PTG) is seen with high centrality of event and involves distress which in this study is represented with posttraumatic stress symptoms (PTSS).

The *negative impact* group consisted of more women that did not get psychological help, experienced their last violence incident more recently (less than 2 years ago), and had lower levels of education, in comparison to *distressed growth* group. Whereas the PTG process requires difficult cognitive processing and the restructuring of schematic structures (Tedeschi et al., 2018), it is logical to assume that when core beliefs are challenged by an IPV experience, psychological assistance helps one through the process required to experience some positive changes (PTG) that are seen in the *distressed growth* group. Psychological help may also be related to the difference in education between these two groups: Robinson et al. (2021) systematic review found that lack of education is one of the factors that creates barriers for seeking help after experiencing IPV. And finally, it is consistent with literature and theory that the *distressed growth* group differs from the *negative impact* group in the length of time since the last violent experience because the PTG process takes time to occur (Despotes et al., 2016; Tedeschi et al., 2018). It can be assumed that for the women in the *negative impact* group experiences of IPV are too fresh and their PTSS manifestation is in its peak. To sum up, the *negative impact* group would likely benefit the most from some additional help or support compared to the rest of the groups in this study. It is possible, that if these women were to receive psychological help, and/or some other assistance in their recovery process, with time some of these women may transition to the *positive growth* or *distressed growth* groups.

The *positive growth* group differs from the *distressed growth* group by time since last violence incident, where women who experienced their last IPV incident more than 2 years ago tended to belong to the *positive growth* group versus the *distressed growth* group. Considering that women in the *positive growth* group experience less PTSS, it is possible that these women have already overcome some of the negative consequences associated with IPV experience. This finding is consistent with Johnson and Zlotnick’s (2012) study, where they found that generally, PTSS decreased over time in IPV survivors, although some women experienced chronic posttraumatic stress disorder (PTSD). However, this result can be viewed in the light of the assumption of a bidirectional relationship between PTSS and centrality of event. It is assumed that the more central traumatic event is, the more it triggers PTSS, and as a result, individuals perceive their traumatic experience to be more central, creating a reinforcing cycle (Boals et al., 2021). In this context, it is possible that women in the *distressed growth* group experience high PTSS because they perceive their IPV experience as more central compared to the *positive growth* group. However, longitudinal data is needed to confirm this assumption. Moreover, the higher levels of resilience in the *positive growth* group have the potential to confirm the theoretical assumptions of PTG process (Tedeschi et al., 2018), where it is assumed that experienced positive changes promote resilience. However, this should be viewed with caution because based on our data we cannot determine if these levels of resilience were promoted by PTG or whether women in this group already had higher levels of resilience prior to their IPV experience.

Finally, the most important difference between the *low impact* and *distressed growth* groups is in the frequency of physical and emotional violence, where more frequent abuse was experienced by women in the *distressed growth* group. According to previous studies, frequent violence is associated with greater PTG (Cobb et al., 2006; Žukauskienė et al., 2019), so it can be assumed that women in the *distressed growth* group experienced more severe IPV (assuming that more frequent violence can be considered as more severe) than in the *low impact* group, and that this severe IPV shattered their core beliefs (centrality of event) and therefore led them to PTG with PTSS and some levels of resilience. If this is the case, then it is logical that women in the *low impact* group are less likely to feel the need to get psychological help. The age differences we found in these groups are consistent with our previous findings (Žukauskienė et al., 2021), and this may imply that younger women have less tolerance for IPV and tend to end the relationship as soon as violence appears, in this way protecting themselves from more traumatic experience.

To conclude, our findings revealed several different patterns in which women undergo their IPV experiences. This lets us draw a rather obvious conclusion, that different women respond differently to IPV, and that even PTG can be experienced in different patterns. These different patterns represent main responses to trauma, where we have women that are suffering greatly from their IPV experience, women that were not affected by their IPV experience, and two groups of women that display two different patterns of recovery from their IPV experience. Also, our results highlight the importance of receiving psychological help, a factor which distinguished women that are experiencing high levels of trauma symptoms from those who also experience positive changes. However, some cultural aspects may be important in women’ help-seeking behavior. Representative survey in Lithuania revealed that 60% of people (men and women) who experienced domestic violence did not seek help (Ministry of Social Security and Labour, 2019). Furthermore, same survey showed that only 55.8% of those who experienced violence knew about institutions helping victims of violence. These numbers indicate that it is not only difficult (for many possible reasons) for a violence survivor to seek help but also, at least in Lithuania, information on the availability of such help does not reach a large proportion of those for who need it most.

Although our results supported some of the theoretical assumptions about PTG and the PTG model, there were some expected patterns we did not find in our study. As described earlier, resilience plays dual role in the model of PTG: Resilience levels can be high before traumatic event that allows to “bounce back” without experiencing growth, and it also can be enhanced after going through PTG process (Tedeschi et al., 2018). However, we did not find a group that has high resilience without greater growth. This could be specific to our sample, but in general, it draws attention to the difficult dynamic between resilience and PTG that merits further investigation.

### Limitations, Future Directions and Implications

Our results should be considered in light of the following strengths and limitations. Although this study uses a person-oriented approach and provides important information about different responses of groups of IPV survivors, it is also a cross-sectional study that cannot capture causality or the directionality of the investigated variables. Another important limitation is that convenient sampling was used, and no record was made of the total number of women asked to participate. This information and more details about the survivors of IPV who refuses to participate in studies like this could give better understanding about the proportion of women that are willing to disclose their experiences and differences between them and those who refuse to participate in studies. Moreover, in the PTG model, distress can be understood broadly and include psychological difficulties such as depression or anxiety, and these difficulties can be also related to other traumatic experiences (e.g., childhood abuse, bereavement, etc.). In this study, we did not measure these variables. Future research should include more indicators of distress and control the impact of other traumatic experiences as this would provide a more complete picture of different responses to IPV.

It should be noted that although Lithuania is becoming increasingly WEIRD (Western, educated, industrialized, rich, and democratic), to some extent study results may be specific to a Northern–Eastern European context. Also, ethnic Lithuanians account for 5/6 of the population, which makes the country one of the most homogeneous in the Baltic States (Statistics Lithuania, 2020), and because of this, there is no stable practice to ask participants about their ethnic background if it is not related to research questions. However, in the context of IPV in Lithuania, this may be an important factor and future research should pay more attention to it.

Among the strengths of the study is the relatively big sample of survivors of IPV. Also, it is one of the few existing studies investigating a theoretical model of posttraumatic growth (PTG), and the only study to our knowledge, that tries to examine the theoretical assumptions presented in the model of PTG in a sample of IPV survivors. Future research should attempt a longitudinal investigation of the PTG model, which would provide the opportunity to capture dynamics among model components that cannot be captured in cross-sectional designs. Specifically, attention should be given to the mechanisms of resilience and PTG as these mechanisms are central to many discussions in the current scientific literature.

The findings of this study indicate that psychological help can be an important resource in helping women recover as positively as possible in their given circumstances. However, other studies in Lithuania show that a large proportion of survivors of domestic violence are reluctant to seek help and are unaware of the possibilities for such help. Policy makers should focus more resources not only on the availability of psychological help to survivors of IPV, but also on better informing and overcoming other barriers (e.g. stigma) that prevents survivors from seeking help.
